# Exploring metal detoxification and accumulation potential during vermicomposting of Tea factory coal ash: sequential extraction and fluorescence probe analysis

**DOI:** 10.1038/srep30402

**Published:** 2016-07-26

**Authors:** Linee Goswami, Sanjay Pratihar, Suman Dasgupta, Pradip Bhattacharyya, Pronab Mudoi, Jayanta Bora, Satya Sundar Bhattacharya, Ki Hyun Kim

**Affiliations:** 1 Department of Environmental Science, Tezpur University, Assam 784028, India; 2 Department of Chemical Sciences, Tezpur University, Assam 784028, India; 3 Department of Molecular Biology and Biotechnology, Tezpur University, Assam 784028, India; 4 Agricultural and Ecological Research Unit, Indian Statistical Institute, Giridih, Jharkhand 815301, India; 5 Department of Civil & Environmental Engineering, Hanyang University, 222 Wangsimni-Ro, Seoul 133-791, Republic of Korea

## Abstract

Metal contamination from coal ashes (CAs) is widely recognized as a significant environmental concern. To learn more about metal detoxification and accumulation potential of earthworm species, metal-rich tea factory coal ashes (TFCA) were fed to *Eisenia fetida* and *Lampito mauritii* by employing a fluorescent tag detection method. Fascinatingly, on feeding fluorescence probed Zn and Cd along with cow dung to *Eisenia fetida*, the detection of the gut-proteins with a molecular mass higher than 100 kDa was a distinct evidence of metal binding. Significant increases were observed in the content of humified organic C [humic acid (HAC) and fulvic acid C (FAC)] and degree of humification during vermicomposting. Concurrently, considerably large amount of toxic metals (Cr, Cd, Pb, and Zn) was transformed from exchangeable to recalcitrant (organic matter and mineral bound) fractions. Moreover, total metal concentrations were reduced with high removal efficiency upon vermicomposting.

Vermicomposting is a mesophilic composting technology that creates a conducive ambience for earthworms and microbes to mineralize complex substances in a complementary manner^1^,^2^. Essential plant nutrients (N, P, K, Ca, Mg, etc) are converted into bioavailable forms, as a substantial part of organic C is stabilized through humification during vermicomposting^3^. These stable, refractory humic substances play an important role in the formation of metal-humus complexes^4^. Nevertheless, little is known about the nature of transformation of metals from exchangeable to obstinate fractions during the vermicomposting processes. Moreover, how or whether the accumulation pathways in earthworm gut influence the levels of metal detoxification is yet inexplicable.

Tea processing units consume considerable amount of coal to produce metal rich coal ashes (CA) in good amount^5^. Various studies reported significant contamination of water, soil, and plant resources with the inflow of toxic metals from CA^6^,^7^,^8^. Recently, we reported the usefulness of vermicomposting technology in stabilizing tea factory coal ash (TFCA) through removal of metals on one hand and increasing nutrient availability on the other^5^. However, the distribution dynamics of metals among exchangeable, oxides, organic matter, and residual fractions (during biodegradation of waste materials) is related to their various nature and bonding strengths^9^. Therefore, the behavior and toxicity of metals during vermicomposting can be assessed further if one knows the chemical forms of different metals involved in the process.

Interestingly, many earthworm species (*Lampito mauritii*, *Eisenia fetida*, *Allolobophora rosea*, and *Nicodrilus caliginosus*) are known to bioaccummulate metals in their intestines by inducing cysteine-rich metal binding metallothioneins^10^,^11^. Metallothionein is a low molecular weight, metal inducible protein that regulates the bioavailability-detoxification dynamics of essential and non-essential metals in earthworm guts^12^. Although mechanism of metal accumulation in earthworms has been characterized repeatedly by assessing metallothionein activity^13^,^14^,^15^, the variability of a response to different nature of feed stocks is still poorly understood.

The use of small molecule fluorescent probes for chemo-selective bio-imaging of living systems is a novel way to navigate biological processes^16^. In this respect, the fluorescence tagging technique is attractive, as one can easily synthesize functional fluorescent reagents that are capable of monitoring intra- and extracellular events with high chemo-selectivity^16^. Hence, ion-induced, metal impregnated fluorescence chemo-sensors may be useful for investigating the synthesis of protein-metal complexes in earthworm intestines with a least possible perturbation to the living system.

In the present investigation, we hypothesized: (a) the formation of humified carbon compounds during the vermicomposting process reallocates the exchangeable forms of metals into recalcitrant forms; and (b) besides metallothionein, there may be yet unknown high molecular weight proteins in earthworm intestine which are capable of sequestering metals. We checked our hypothesis by using fluorescent chemo-sensors coordinated with Zn and Cd for the first time to elucidate the synthesis of protein-metal complexes in the earthworm intestines with the least disruption to the living system. Moreover, the metal-carbon dynamics have been assessed temporally through the apportionment of various metal fractions and humification attributes.

## Results

### Changes in C/N ratio, Compost respiration (CR), humic acid carbon (HAC), fulvic acid carbon (FAC), and degree of humification (DoH)

The changes in C/N ratio and CR are presented in Table 1. Table 2 presents the data on HAC, FAC, and DoH in the various feed mixtures during the biocomposting process. After 60 days of incubation, C/N ratio values were reduced approximately from 2.48 to 4.69 folds in *Eisenia* vermibeds and approximately from 2.13 to 7.64 folds in *Lampito* vermibeds. However, the extent of reduction was less conspicuous in aerobic composting as compared to vermicomposting. A significantly low C/N ratio was recorded under E1, L2 and L4 followed by E3, L1, E2, E4 and L3 (P for treatment = 0.000; LSD: 1.04). Concurrently, a significant rise in CR was observed in both *Eisenia* and *Lampito* vermicomposting systems between 0 and 30 days, indicating the maturity of the compost. Interestingly, CR slightly receded with vermicomposting in between 30 and 60 days (Table 1). In contrast, we found a gradual rise in CR with the composting system until the 60^th^ day of incubation, although the magnitude of CO_2_ evolution (i.e. CR) was generally higher in vermicomposting systems than in aerobic composting. A significantly high CR (Table 1) was recorded in L3 followed by L4, E4, E2, and L2 at the end of the incubation period (P = 0.000; LSD = 0.18). Interestingly, Both the C/N ratio and CR are vital indicators of compost maturity and quality.

The humified OC from both fulvic acid carbon (FAC) and humic acid carbon (HAC) represents the relentless pool of OC with a mean residence time of several hundred years; they also act as a stable sink for metals. Hence, the increment in HAC and FAC may reflect the stabilization of the composting process and storage of organic C. Interestingly, the content of both FAC and HAC increased considerably with vermicomposting, whereas their aerobic composting counterparts decreased slightly in all the TFCA containing feed mixtures after 30 days (Table 2). A noticeable increase in FAC was recorded in E3, followed by E4, L3, L2 and L8 (P value 0.000, LSD 0.303). On the other hand, the content of HAC in the finished product was seen in the following order: E3 > E2 > L4 > L2 = L3 > E4 > C4 > L1 = E1 > C1 = C2 = C3 (P value: 0.000 and LSD: 0.12) (Table 2).

The degree of humification (DoH) can be a meaningful barometer to express the ratio of aromatic and aliphatic C compounds in the biocomposting system. The DoH values also increased significantly over time under both types of vermicomposting systems (Table 2). The production of humified substances was considerably higher in the *Lampito* vermicomposting (L3 and L4) system as compared to the *Eisenia* mediated system (E2, E3, and E4) (P value = 0.000; LSD = 0.16). Moreover, according to the two-way ANOVA, the treatment × day interaction was highly significant for HAC, FAC, and DoH (P values for treatment × day = 0.000) (Table 2). This indicates that the effect of the composting period was equally significant along with the treatment type.

### Dynamics of metal fractions during bioconversion of TFCA

**Figure 1:**
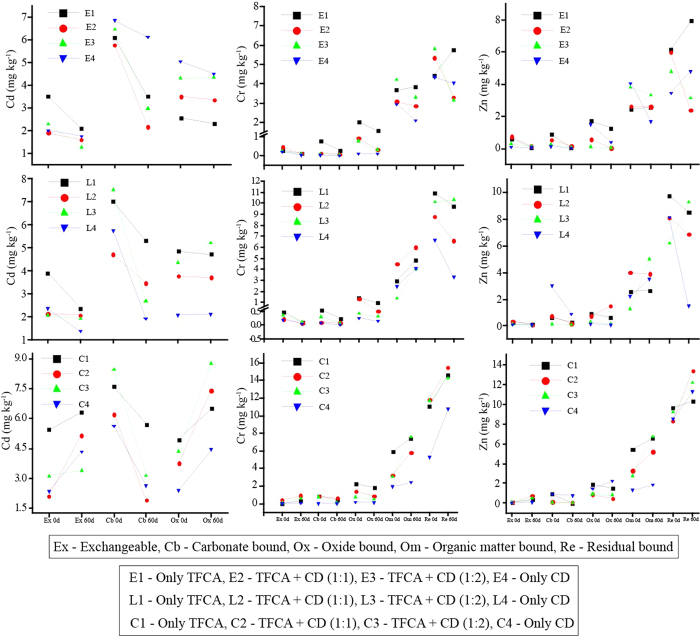
Temporal changes in various fractions of Cd, Cr, and Zn during composting and vermicomposting (mean ± standard deviation) with the LSD (the least significant deviation).

Metals in composts are generally present in various forms, easily exchangeable or recalcitrant ones, as they can freely interact with various components (like oxides, carbonates, organic matter, and residual mineral fractions). The process of composting can proceed toward either immobilization or dilution of metals present in the feed stocks. On the other hand, earthworms play a vital role in immobilization of metals via vermicomposting. The temporal trend of apportionment in different forms of metals (Cd, Cr, and Zn) between three bioconversion systems (*Eisenia* vermicompost, *Lampito* vermicompost, and aerobic compost) with TFCA-based feed stocks is plotted in Fig. 1. The detailed results for all six metals are also presented in Supplementary information (Table 1S (a–j)). In the case of Zn, Cr, and Cd, the exchangeability decreased significantly under vermicomposting conditions over time (P for day & treatment × day = 0.000) (Fig. 1; Supplementary information Table 1Sa to f). The lowest content of Zn in exchangeable fraction was observed in E4 and L4 (P = 0.000; LSD = 0.05) (Fig. 1; Supplementary table 1Sc). The bioavailability of Cr was also at the lowest level in E3, L3, and L4, whereas the lowest level of bioavailable Cd was seen in E3, E4, and L4 (P = 0.000; LSD: Cr = 0.06; and Cd = 0.25) (Fig. 1; Supplementary information Table 1Sc and 1Se). The highest reduction of Cd occurred in samples E4 (54.44%) and L4 (37.13%), as compared to the initial values.

It is interesting to find that oxide and CO_3_^2−^ bound fractions of Cr and Cd were reduced significantly with both the vermicomposting systems (Fig. 1) (P = 0.000). Additionally, highly immobilized metal fractions (organic matter and residual bound) considerably increased with vermicomposting, whereas the bioavailability of all the metals remarkably increased in almost all types of feed stocks subjected to aerobic composting (P = 0.000). However, Cd was not detected in the organic matter or residual fractions from the three composting systems (Supplementary information Table 1Se).

Among the six metals, arsenic was detected only in exchangeable form in all three systems during the study period (Supplementary information Table 1Sf). In the present investigation arsenic concentrations were significantly reduced during the period of incubation under both the vermicomposting systems. The lowest As bioavailability in E2 was followed by L1 and L4 at the end of the study (P = 0.000; LSD = 0.03). Contrary to arsenic, the fractionation patterns of other metals (Fe, Cr, Zn, Pb, and Cd) were highly variable during the bioconversion process. A significant reduction of Fe in both exchangeable and organic/residual fractions was seen in *Eisenia* vermicomposts (E2, E3, and E4) over time (P for treatment & day = 0.000) (Supplementary Information Table 1S g and h). On the other hand, organic matter and residual fractions of Fe increased substantially under aerobic composting (P for treatment & day = 0.000) and highest allocation of Fe in the residual fraction was recorded in C2 followed by C3 and C4 (LSD = 110.05).

A similar trend of reduction in the exchangeable fraction was also seen in Pb with vermicomposting (Supplementary Information Table 1S i) (P value: treatment = 0.000; day = 0.000; and treatment × day = 0.000). The levels of Pb were reduced considerably in E3, E4, and L1 at the end of the incubation period (LSD = 0.56). In general, except As and Cd, all other metals exhibited a considerable increase in organic matter and residual fractions during the bioconversion process (Fig. 1; Table 1S: b (Zn), d (Cr), h (Fe), and j (Pb)). As such, there was a significant increase in the formation of stable humic substances (FAC and HAC) and dominance of aromatic C compounds for all three composting processes. Under such circumstances, transformation of metals from bioavailable forms to highly recalcitrant forms should have been favored.

### Total metal concentration, removal efficiency, and metallothionein (MT) content in earthworm gut

**Figure 2:**
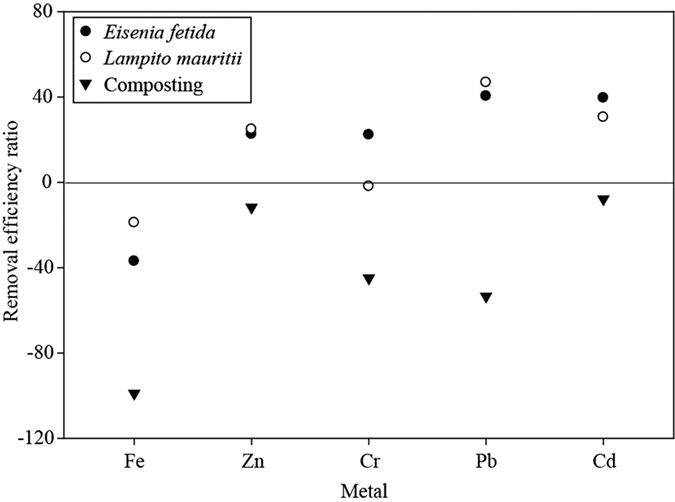
Metal removal efficiency of the three bio-composting systems within 60 days.

**Figure 3:**
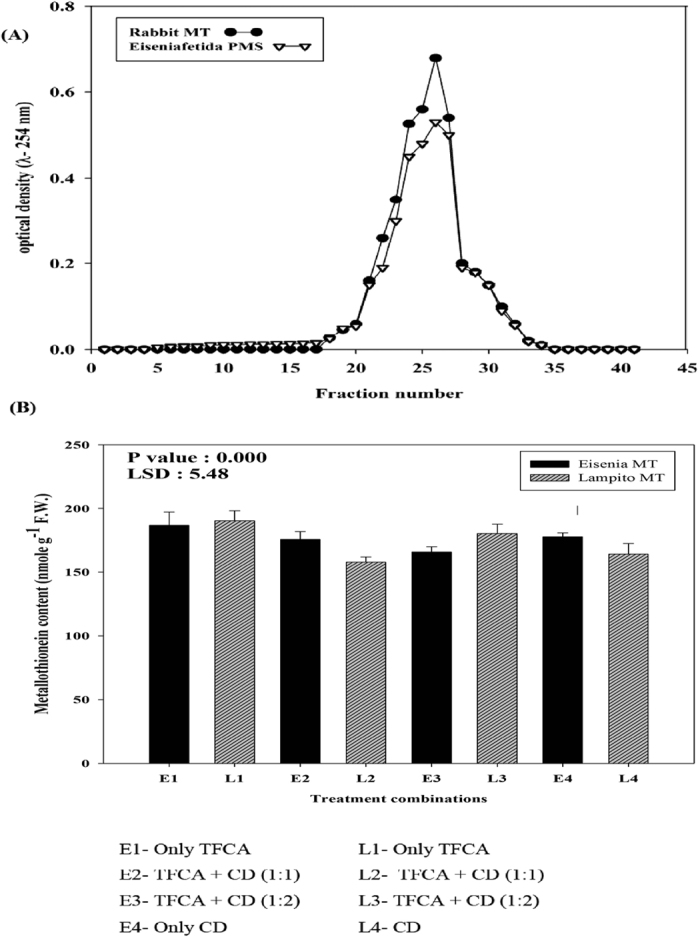
(**A**) Sephadex G-75 elution profile of *Eisenia fetida* and purified rabbit liver metallothionein (MT) [Eluted in 50 mM Tris-HCl buffer (pH 7.4), column size 25 × 1.5 cm, and flow rate 35 ml h^−1^]; and (**B**) Metallothionein content in *Eisenia fetida* and *Lampito mauritii* during vermicomposting with various Tea factory coal ash (TFCA) and cow dung (CD) mixtures (Error bars represent the standard deviation of three replicates).

A substantial allocation of Fe was observed in the bound fractions (organic matter and residual) during the later stage of vermicomposting. Its total concentration, however, increased at the end with both vermicomposting and composting (Table 2S). Hence, the removal efficiency of Fe was negative in all three composting systems (Fig. 2). However, Fe is an essential element for plants and animals. Interestingly, we observed a noticeable reduction in the total concentration of Zn, Cr, Pb, and Cd at the end of the incubation period in vermicomposts (Tables 2S and 3S) with P (Fe = 0.000, Zn = 0.004, Cr = 0.021, Pb = 0.003, and Cd = 0.000) and LSD values (Fe = 843.2, Zn = 2.42, Cr = 2.22, Pb = 0.72, and Cd = 2.93). Moreover, significant variations were seen in time scale with the evidence of interactions between time and concentration of metals (Zn, Cr, Pb, and Cd) in various feed stocks (P = 0.000 for both day and treatment × day). In contrast, all of those metals increased considerably under aerobic composting. Hence, the removal efficiency (RE) of different metals was compared between all three types of composting systems (Fig. 2). Interestingly, a high RE of Zn, Cr, Pb, and Cd was recorded for *Eisenia fetida* and *Lampito mauritii. E. fetida* was highly efficient in removing Cr and Cd from the feed mixture, whereas Pb accumulation potential was higher in *L. mauritii* than *E. fetida* (Fig. 2). Presently, the enhanced bioaccumulation efficiency of *E. fetida* and *L. mauritii* should have favorably reduced the total content of metals other than Fe (Cr, Zn, Pb, and Cd) in the vermicompost. The lucid evidence of high efficiency in metal removal by the two earthworm species encouraged us to explore the underlying mechanism of this phenomenon. Here, we rechecked the validity of our previous findings (cited earlier) by conducting another round of MT radio assay (Fig. 3). Significantly high MT content was observed in both species when fed with only TFCA (L1 and E1) followed by only CD for *E. fetida* (E4) and TFCA + CD (2:1) for *L. mauritii* (L3) (P = 0.000; LSD = 5.48).

In this experiment, the ^203^Hg incubated post mitochondrial supernatant (PMS) of the whole body tissue for *Eisenia fetida* was prepared; the specificity of ^203^Hg saturation by MT was checked by recording radioactivity of the collected fraction in the scintillation counter. Interestingly, we found clear resemblance in saturation peak of MT in the eluted fractions of *E. fetida* with the MT protein identified for rabbit liver (commercial) through chromatographic analysis (Fig. 3). However, it was not supported statistically (e.g., correlation analysis) with respect to MT content in earthworm intestine and metal (Zn, Cu, Pb, and Cd) concentration in the feed stock (Table 4S). Although the highest correlation co-efficient for MT (in terms of the Pearson’s r value) was obtained with Cr (0.61) followed by Zn (0.41), all values were insignificant in terms of probability of correlations (P > 0.1). Consequently, there may be some other metal binding biomolecules in the earthworm gut that can play a vital role in metal sequestration along with MT. This hypothesis has been substantiated in the following section.

### Metal accumulation in earthworm intestine: A fluorescence probe and histology based study

**Figure 4:**
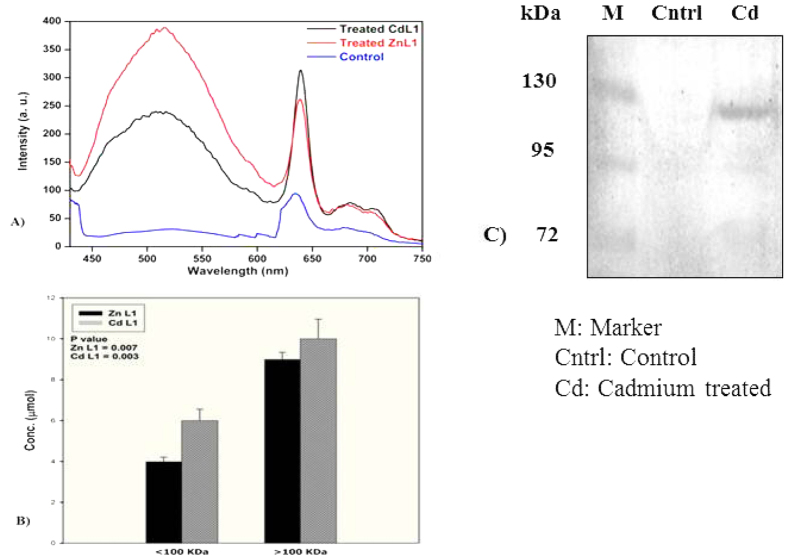
(**A**) The fluorescence emission peaks of Cd and Zn-ligand complex treated (Treated CdL1 and Treated ZnL1) and untreated (control) earthworm gut protein (above 100 KDa); (**B**) The accumulation concentration (μM) of CdL1 and ZnL1 in <100 KDa and >100 KDa protein complexes in the earthworm intestines; and (**C**) Western Blot analysis of SDS-PAGE resolved earthworm protein (EP) using anti-EP antibody.

**Figure 5:**
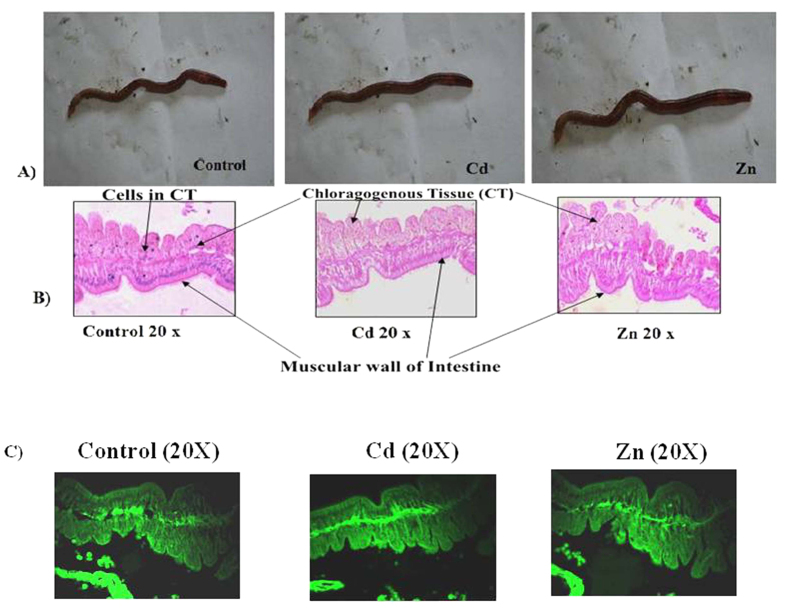
(**A**) External morphological photographs of the treated *Eisenia fetida* specimens; (**B**) Histology of the intestine of *Eisenia fetida* treated with Cd (Cd L1) and Zn (Zn L1) complex compared to control specimen and (**C**); and Fluorescence microscopy of histological information of treated and untreated *Eisenia fetida* intestines.

The results of this study demonstrated that the exchangeable fractions of metals shifted towards obstinate ones, and that their total concentrations were reduced upon vermicomposting. Therefore, the possibility of the existence of some metal binding non-metallothionein proteins in the earthworm guts cannot be overruled. In order to verify such a premise, the fluorescence-tagged Cd and Zn were added to the cow dung (CD)-based vermibeds of *Eisenia fetida* and the protein complex of high molecular mass was targeted for assessment (Fig 4a). In our samples the emission peaks of the treated samples clearly confirmed the signature of the fluorescence-tagged metal compounds bound with proteins of a molecular mass greater than 100 kDa (Fig 5a). Interestingly, a similar peak between 450 to 550 nm wavelengths of the fluorescence spectrum could not be detected in the control samples. The emission peaks at 640, 680, and 710 nm in both control and treated samples should probably be ascribed to some other fluorescent biomolecules that are present in the earthworm guts. Moreover, high concentration of Zn and Cd complexes were detected in >100 kDa as compared to the <100 kDa proteins (Fig 4b). Extract from gut epithelial cells of earthworm was subjected to molecular weight cut. Then, proteins with >100 kDa were collected and passed through sephadex G-100 column. Afterwards, the protein fraction with Cd induced expression was assessed in comparison to the control. This sample was subsequently lyophilized and subjected to SDS PAGE for immunoblot analysis by using antibody raised against this protein in mice (Fig 4c). As seen in Fig 4c, it is striking to find that the molecular weight of this protein was approximately 130 kDa. In light of the fact that the molecular weights of metal bind proteins (metallothioneins) or peptides (phytochelatins) are low, this newly detected protein is likely to exhibit unique characteristics with a possibly significant role in binding toxic metals.

Morphologically, there was no sign of any dryness or damage to the dermal portion of the earthworm due to the exposure to Cd and Zn (Fig. 5A). Moreover, histological analysis of chloragogenous tissues in the gut of control and treated *E. fetida* clearly indicated that there was no degeneration or abnormality in the tissues due to these metals, as long as the present experimental conditions were concerned (Fig. 5B,C). However, we could not find any evidence of damage to the intestinal wall of *Eisenia fetida* in our experiment.

## Discussion

Based on our hypothesis stated end of in the introduction, the major objectives of this research can be classified as: a) to use the fluorescence chemosensors of Zn and Cd in the quest of an unknown pathway for metal binding by earthworms and b) to monitor the apportionment of exchangeable forms of metals to recalcitrant ones and to relate the transformation with humification process of organic matter.

It has long been known that in many organisms the physiological tolerance to heavy metals is attributed to induction of metal chelating proteins^17^. These metal chelating proteins, known as metallothioneins (MTs), are low in molecular weight and rich in cysteine. Earthworms are also known to thrive in metal contaminated soils because of their ability to bind high concentrations of heavy metals in their tissues. Past researches have revealed at least two coexisting metal binding mechanisms in earthworms^18^. Primarily, the metals are retained in insoluble calcium phosphate granules or chloragosomes^17^. As a result, the metals remain insoluble and cannot influence the normal biochemical processes in the cytoplasm^17^. Later, the insoluble metals are chelated by the sulphur donating ligands of MTs and are transported to the chloragogenous tissues of the intestines, where they are neutralized^18^. This pathway has also been vindicated with formidable evidence in a recent report^11^.

In the present experiment, we also observed significantly high content of MT in *E. fetida* (187 nmole g^−1^) and *L. mauritii* (190 nmole g^−1^) upon exposure to metal rich TFCA (P value: 0.000 and LSD = 5.48) (Fig. 3). However, earthworms in soil ecosystems and earthworms in vermicomposting reactors may or may not behave identically^19^. In recent years, several researchers reported the possibility that heavy metal concentrations increase during the vermicomposting process^20^,^21^,^22^. Nonetheless, no valid rationale has been supported by experimental evidence. On the other hand, many studies have reported significant reduction in heavy metal concentrations during vermicomposting^2^,^5^,^23^. There are two probable reasons behind such phenomena. Firstly, the earthworm mediated biodegradation process increases the levels of humic fractions, which can strongly immobilize metals by the formation of stable metal-humus complexes^4^; secondly, the earthworms bind metals through the well-known pathway discussed earlier^11^. Nevertheless, a lot of ambiguities still persist because the metal binding ability of earthworms is often inconsistent^2^,^9^,^23^. The differential nature of metal solubility may be an important factor in this regard. For example, Cd and Zn are more soluble than Pb, Cr, or Cu^24^. Interestingly, our present findings also demonstrated the absence of Cd in organic matter or residual bound fractions. We were also unable to detect any significant correlations between MT content in earthworm guts and metal concentration in vermicasts (P > 0.1). Above all, there may be some unknown mechanism of metal binding in earthworms which may also vary between species. At this juncture, the fluorescence probed metals (Cd and Zn) could facilitate exploring a new pathway which we propose in this communication.

Over the course of this study, the accumulation/detoxification potential of metals was investigated using the vermicomposting system in comparison to aerobic composting. The results of this research successfully demonstrated the variability in fractional as well as total concentrations of metals during the various types of composting processes (aerobic composting, *Eisenia* vermicompost, and *Lampito* vermicompost). If the organic matter is to be degraded under aerobic composting conditions, the volume of substrate can be reduced greatly. Consequently, an increase in the total content of metals is accompanied inevitably in the end product^25^. Likewise, Song *et al*.^2^ reported a significant increase in the total metal concentration on vermicomposting. They argued that accelerated decomposition of feed stocks with vermicomposting resulted in a rise in the total metal content. However, in this study, the contrasting trend may be explained by the formation of stable humic substances in the processed vermicomposted products, which should have facilitated the allocation of metals in organic matter and/or residual bound fractions. In addition, the overall removal efficiency of the target metals was higher under vermicomposting conditions with *E. fetida* than with *L. mauritii*.

Quite a large fraction of metals was changed from exchangeable to oxide, carbonate, organic matter, and residual bound fractions during vermicomposting as well as composting of various TFCA and CD mixtures. The exchangeable fraction was the readily bio-available form, whereas those bound with organic matter was the recalcitrant fraction of all metals^9^. Hence, we may be able to justify our hypothesis by citing the cases for these two fractions with statistical validity. The exchangeable fractions of Zn were reduced by 1.7–7.1 folds in *Eisenia* system, while 1.6–5.7 folds in *Lampito* system (P = 0.000 and LSD = 0.054) (Table 1a). In contrast, such a reduction for Cr was 1.6–3.2 folds in *Eisenia* system and 2.5–7.2 folds in *Lampito* system (P = 0.000 and LSD = 1.06) (Table 1c). A significant reduction in bio-availability was also recorded in E3 [TFCA + CD (1:2)_*Eisenia*_] followed by L4 [CD only _*Lampito*_] (P = 0.000 and LSD = 0.25). On the other hand, organic matter bound fraction of Zn was significantly enhanced in the *Lampito* system (1–4 folds) and aerobic composting system (1.2–1.5 folds) (P = 0.000 and LSD = 0.034). Similar evidence was also found for Cr in all the three biocomposting conditions (P = 0.000; LSD = 0.027) (Table 1d). At the same time, evidence was obtained regarding the formation of stable humic fractions for organic C. A significant increment in HAC (1.12–2.47 folds) and FAC (1.4–27 folds) was noted during vermicomposting (P for treatment & day = 0.000), whereas the degree of humification markedly increased by 3.1–8 folds in the vermicomposts (P for treatment = 0.000). This is interesting because the improvement in degree of humification indicates the formation of highly stable aromatic compounds which have great potential for metal sequestration^3^,^4^.

The bound forms of As was not detected in this experiment. Generally, elemental (As) and oxide (As_2_O_3_) forms are the most abundant species of arsenic emitted from the coal combustion atmosphere^7^. However, the oxide forms of As are less likely to occur in the TFCA residues because they are often volatilized under low temperature combustion conditions in the tea factories^5^. This may be due to loss in weight during the process of composting, while loss of C is known to proceed through mineralization^9^. While it was reported that Cd condensation was influenced by the aeration status of the composting process and the properties of feed stocks^26^,^27^. Hence, the allocation of Cd in recalcitrant fractions was probably affected by the constant air circulation within the system as both the composting systems were aerobic.

Overall, a steady decline in total metal content in the vermicomposted products suggested that a considerable amount of metals may be accumulated by the earthworms. It is well known that earthworms accumulate metals using metallothionein (MT), a group of cysteine-rich, low molecular mass proteins, in their intestines^5^,^17^. The molecular mass of metallothionein was reported to be lower than 50 kDa^14^,^28^. In our previous report, the MT expression was observed in *E. fetida*^5^. Interestingly, in the specimens used in this study, the MT expression was again verified. Moreover, the results were consistently in good agreement with our previous findings^5^. Recently, Liebeke *et al*.^29^ proposed a new pathway of metal chelation for a small oligomer, phytochelatin in the earthworm guts. Generally, phytochelatins are present widely in various plants. This remarkable coincidence encouraged us to explore the fundamental features of metal binding phenomenon in this earthworm species.

In this study, we investigated a new mechanism of metal detoxification in the intestines of *Eisenia fetid*a by employing fluorescent chemosensors of Cd and Zn. Application of Cd and Zn fluorescent ligand complexes in the cow dung-based vermibeds of *E. fetida* revealed that the metal-protein complexation proceeded to a large extent in earthworm intestines. Moreover, the results of molecular exclusion chromatography indicated the existence of some metal binding proteins in *E. fetida* with high molecular weight as they are over expressed relative to untreated earthworm. We have thus provided the existence of one of such proteins through immunoblot. These unidentified metal binding proteins with high molecular weight in earthworm gut should offer a new area of research to learn about the earthworms’ potentiality to minimize heavy metal toxicity.

The histological study of the treated and control earthworm specimens (Fig. 5) also exhibited interesting outcome. It was reported previously that the exposure to Cd should have caused significant damage to the intestinal wall of *Eudrillus euginae*^30^. Contrary to such finding, we were unable to find such abnormality in *Eisenia fetida*. There may be two reasons to explain such differences between the studies: (a) our predecessors used substantially high concentrations of metals (50–1000 mg kg^−1^), while they were exposed to considerably low concentration in this work (50 mg kg^−1^) and (b) the duration of our study was also shorter (60 days) than that of their experiment. *Eisenia fetida* is known to have greater tolerance to metal exposure than many earthworm species^31^. Moreover, exposure history of the stock population is likely to influence the metal tolerance of the organisms^32^.

## Experimental

### Collection of TFCA and earthworms: experimental set up

Well characterized TFCAs needed for the preparation of experimental beds were collected from Sonitpur, Assam, India^5^. The general composition of TFCA was: pH - 5.9; bulk density - 0.58 g cc^−1^; water holding capacity - 75%; Total organic C - 2.8%; Available N - 30.5 mg kg^−1^; Available P- 51.5 mg kg^−1^; Available K - 65.7 mg kg^−1^; Exchangeable- Fe - 12.3 mg kg^−1^; Zn - 0.12 mg kg^−1^ ; Cr - 0.32 mg kg^−1^; Cd -1.1 mg kg^−1^; and As - 0.12 mg kg^−1^. Clitellated, adult earthworm (*Eisenia fetida* Savigny and *Lampito mauritii* Kinberg) specimens, weighing about 300–400 mg, were collected from the vermiculture unit of the department of Environmental Science, Tezpur University, Assam and used for this study.

### Design of Bioconversion systems

Experimental beds were prepared for three bioconversion systems (one aerobic composting and two types of vermicomposting systems employing *Eisenia fetida* and *Lampito mauritii*). Prior to incubation, TFCA and CD were thoroughly mixed in various combinations. Afterwards, each mixture, in triplicates, was poured in perforated earthen vessels (3 L capacity) and kept undisturbed for a week for softening of wastes and thermo stabilization. Subsequently, gut evacuated adult earthworm specimens (6–7 cm length) were inoculated in the vermiconversion systems @ 10 worms’ kg^−1^ of the feed mixtures. The study was conducted for 60 days during the late monsoon season (August–September) of 2014. We uniformly maintained 40–50% moisture by sprinkling deionized water at 2–3 days of interval. The substrates were churned daily for 30 minutes throughout the incubation period (60 days). The ambient temperature during the study period was recorded between 28 °C to 32 °C. The following combinations of TFCA and CD were selected for the three bioconversion systems:[Table t3]

Samples were drawn periodically at an interval of 30 days starting from the day of commencement (i.e. 0 day) of the experiment; and analyzed for various attributes as described in the following sections.

### Analysis of carbon fractions, degree of humification, and compost respiration

The compost respiration (CR) was evaluated following the procedure of Epstein *et al*.^33^. HAC, FAC, and DoH were determined sequentially using the sodium pyrophosphate extraction procedure of Page *et al*.^34^. Initially, concentrated H_2_SO_4_ was added to the sodium pyrophosphate extracts and kept overnight. In the next day, the filtrates were titrimetrically analyzed for FAC and the remaining precipitates were washed with 0.1 N NaOH. Subsequently, 5 ml of the washed solutions were taken for the analysis of HAC using a titration method. The remaining solutions were taken for enumerating the aliphatic and aromatic hydrocarbons in a UV-VIS spectrophotometer at 465 and 665 nm, respectively to calculate the DoH as defined below:


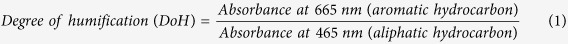


### Sequential extraction of metals and total metal concentration

Chemical fractionation of metal (As, Fe, Cr, Cd, Pb, and Zn) forms in bio-converted samples was carried out following the procedure of Tessier *et al*.^35^. 1.0 g of each sample (in dry weight) were taken in 50 ml conical flasks, and the following fractions were sequentially obtained:

1. Exchangeable fraction: Samples were extracted at room temperature with 1M MgCl_2_ by shaking at 120 rpm for 1 h.
2. Carbonate bound fraction: The residue from [1] was extracted with 1M CH_3_COONa by shaking at 120 rpm for 1 h.
3. Fe-Mn bound fraction: The residue from [2] was extracted with 20 ml of NH_2_OH.HCl by shaking (120 rpm) at 96 °C for 6 hr.
4. Organic matter bound fraction: To the residue from [3], 5 ml of H_2_O_2_ (30%) were added (along with 0.02 M HNO_3_) and heated at 85 °C for 2 h with occasional agitation. Again, 3 ml of H_2_O_2_ (30%) were added to prevent foaming and the heating was continued for another 3 h. After cooling, 3.2 M NH_4_COOCH_3_ was added and the samples were diluted to 20 ml and agitated continuously for 30 minutes.
5. Residual fraction: Concentrated HNO_3_ was added in the residue from step [4] and heated at 105 °C to dry the contents. The dried samples were further diluted to 25 ml.

Concentrations of metal species (As, Fe, Cr, Pb, Zn, and Cd) in each extract were analyzed in ICP-OES. The results of all the fractions were summed up to obtain the total concentrations of each metal analyzed as follows:


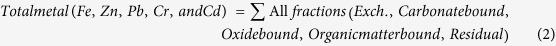


### Metal removal efficiency

To compare the potential removal efficiency (RE) of the metals between three bio-composting systems [(1) *Eisenia* vermicompost, (2) *Lampito* vermicompost, and (3) aerobic compost], the total metal concentrations were computed as shown above at the start (t = 0 day) and the end (t = 60 days) for each experiment and the RE values were computed using the following equation^23^:


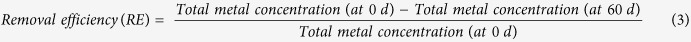


### Chemicals and materials

We procured the multi-element stock solution of As, Fe, Cr, Pb, Cd, and Zn (1000 ± 2 mg L^−1^ in 5% HNO_3_) from Merck. The stock solution was diluted with 0.1 M HNO_3_into five different levels for preparing the calibration standards. We used deionized water and Suprapur acids for preparing all analytical solutions. The precision of the ICP-OES method was verified in regard to relative standard deviation (RSD) and the results of As, Fe, Cr, Pb, Cd, and Zn were 4.3%, 5.2%, 4.5%, 4.8%, 8.6%, and 2.8%, respectively. The detection limits in ICP-OES for the studied metals were computed as: As = 0.01 mg L^−1^; Fe = 0.01 mg L^−1^; Cr = 0.01 mg L^−1^; Pb = 0.01 mg L^−1^; Cd = 0.02 mg L^−1^; and Zn = 0.01 mg L^−1^.

### Fluorescence probes of Zn and Cd: Chemistry and utility

#### Synthesis of Ligand (L1)

The ligand (L1) was synthesized through condensation reaction between 2-amino 6-methyl pyridine-2-amine (30 mmol) and 2-hydroxy benzaldehyde (30 mmol) in presence of toluene. After the completion of the reaction, a yellow condensed product was obtained which was dried under reduced pressure and used for further analysis. Yield = 5.6 g (88%). IR (KBr, cm^−1^): ν = 3058, 2969 (C–H), 1620 (C = N). δ_H_(400 MHz; CDCl_3_): 2.58 (s, 3H, CH_3_), 6.96 (t, 1H, *J* = *7.5* *Hz*), 7.03 (d, 1H,J = 8.0 Hz), 7.12 (d, 1H,J = 7.8 Hz), 7.15 (d, 1H, J = 7.8 Hz), 7.40 (d, 1H, J = 7.8 Hz,), 7.52 (d, 1H, J = 7.6 Hz,), 7.70 (t, 1H, J = 7.8), 9.47 (s, 1 H, HC = N), and 13.52 (s, 1H, OH).

#### General procedure to synthesize the complexes

The complexes of Zn(II) and Cd(II) with ligand (L1) were synthesized using the reported procedure of Enamullah *et al*.^36^. The ZnL1_2_ complex was synthesized from reaction between Zn(O_2_CCH_3_)_2_.2H_2_O and L1 in the presence of NaHCO_3_ in refluxing methanol [Yield = 0.430 g (83%). IR (KBr, cm^−1^): ν = 3070, 2969 (C–H), 1614 (C = N). Δ _H_(400 MHz; CDCl_3_): 2.32 (s, 3H, CH_3_), 6.72 (t, 1H,*J* = *7.6* *Hz*), 6.93 (d,1H,*J* = *8.0 Hz*), 7.0 (d, 1H, *J* = 7.5 *Hz*), 7.06 (d, 1H, *J* = *7.6* *Hz*,), 7.32 (t, 1H, *J* = *7.8* *Hz*), 7.45 (d, 1H, *J* = *7.5* *Hz*), 7.55 (t, 1H, *J* = *7.5* *Hz*), and 9.4 (s, 1H)]. Whereas, the Cd (L1)_2_ complex was synthesized through a reaction between CdCl_2_ and L1 in 1:2 ratio in the presence of NaHCO_3_ in refluxing methanol [Yield = 0.480 g (85%). IR (KBr, cm^−1^): ν = 3075 (C–H), 1610 (C = N). δ_H_(400 MHz; CDCl_3_):2.30 (s, 3H, CH_3_), 6.68 (t, 1H,*J* = *7.8* *Hz*), 6.90 (d,1H,*J* = *7.8* *Hz*), 7.03 (d, 1H, *J* = *7.6* *Hz*), 7.08 (d, 1H, *J* = *7.8* *Hz*,), 7.34 (t, 1H, *J* = *7.6* *Hz*), 7.43 (d, 1H, *J* = *7.6* *Hz*), 7.56 (t, 1H, *J* = *7.8* *Hz*), and 9.5 (s, 1H)].

#### Spectroscopic studies

**Figure 6:**
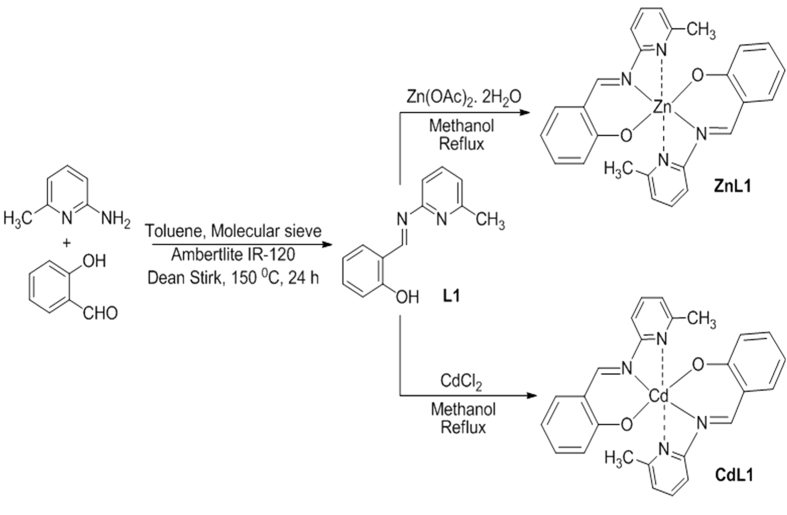
Synthetic route of zinc (ZnL1) and cadmium (CdL1) complexes with the ligands.

**Figure 7:**
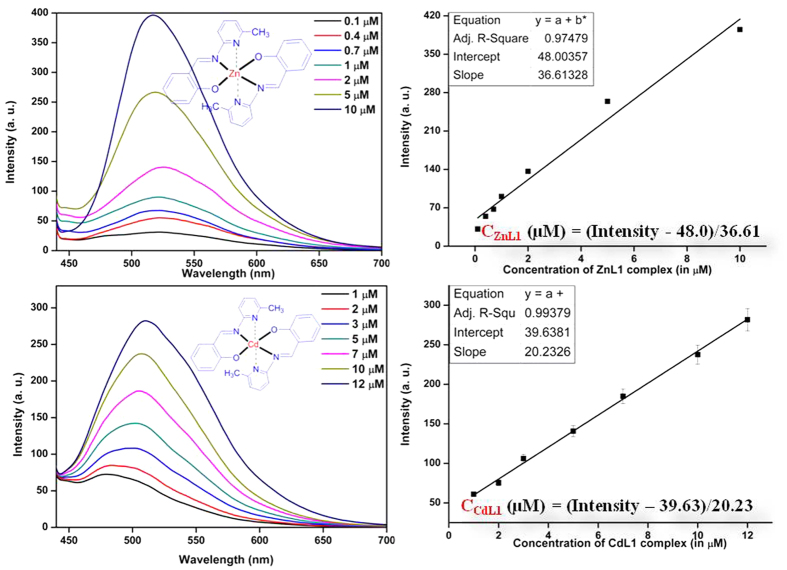
Emission spectra of ZnL1 and CdL1 complexes and relationship between their emission intensity versus concentration plot.

The synthesized Schiff base ligand L1 showed three peaks in DMSO (at 263, 312, and 342 nm) with extinction coefficients (ɛ, mol^−1^ L cm^−1^) of 19,600, 21,500, and 20,600, respectively. Upon complexation of ligand with Zn (II) (ZnL1), one new peak appeared at 420 nm with extinction coefficients (ɛ, mol^−1^ L cm^−1^) of 12,345, assigned to the metal to ligand (ML) charge transfer transition. On the other hand, complex CdL1 exhibited two peaks at 310 and 422 nm with extinction coefficients (ɛ, mol^−1^ L cm^−1^) of 11,285 and 12,545, respectively, for intra ligand and metal to ligand (ML) charge transfer transition. Upon excitation of both the ZnL1 and CdL1 complex (at 412 nm), one unique new broad emission band (around 450–650 nm) appeared from both the complexes. We subsequently chose 510 to 520 nm emission wavelengths for ZnL1 and CdL1, respectively, to measure the intensity of the calibration curves of both the complexes (Fig. 6). The emission intensity of both complexes, obtained across different concentration levels, was plotted to yield two different equations for ZnL1 and CdL1 (Fig. 7).Based on these two equations one should be able to detect and estimate the concentrations of both the complexes in an unknown sample at micromolar (μM) levels of concentration.

#### Metallothionein (MT) radio assay

Prior to the fluorescence probe analysis, we carried out MT radio assay in a Perkin Elmer Scintillation counter (Model 2800) using ^203^Hg as the biomarker. For this, we followed the procedure of Kotsonis and Klaassen^37^ as described in details in our previous publication^5^. However, unlike our previous experiment, here earthworms were collected after 60 days of incubation, gut cleaned, freeze killed, and used for the study. The accuracy of MT radio assay was reconfirmed through gel filtration chromatography as described by Maity *et al*.^10^ and Goswami *et al*.^5^.

#### Metal accumulation in Eisenia fetida through Zn L1 and Cd L1 probe and histological analysis

Non-clitellated juvenile *Eisenia fetida* specimens were collected from the vermiculture unit of the Department of Environmental Science, Tezpur University and reared in urine-free cow dung for two weeks. Aqueous solutions @ 10 ml kg^−1^ of fluorescent-labeled Cd (CdL1) and Zn (ZnL1) (Concentration: 5 mg ml^−1^) were added individually to the feed stock and then incubated for 2 months. After incubation, earthworms were collected and kept overnight in moist filter paper for gut cleaning. Gut cleaned, freeze killed earthworms were sonicated and the homogenate sample was centrifuged at 10,000 rpm for 15 min. Supernatants were collected and subjected to Amicon YM-50 and YM-100 filter devices (Millipore, Bedford, MA) for separating the proteins based on their molecular weight. The filtrate and retentate from these filter devices were collected, and the fluorescence in the separated protein samples was analyzed using a fluorescence spectrophotometer (Perkin Elmer; Model: LS 55). The concentration of CdL1 and ZnL1 was measured using the standard curve as shown in Fig. 6.

Simultaneously, another set of gut cleaned earthworms were collected after 60 days and killed by freezing, washed with de-ionized water, and used for histological analysis by adopting the procedure of Sharma and Satyanarayan^30^. Briefly, the earthworm tissues were fixed in Bouin’s fluid for 24 hrs and dehydrated in graded alcohol from 30% to 100% followed by xylene for 10 minutes and embedded in paraffin. Sections of 5 μm thickness were cut with the help of a microtome and mounted on albumin coated slides. The slides were then subjected to routine hematoxylin-eosin staining and analyzed in a high resolution microscope.

#### Electrophoresis and Western blotting

Earthworm gut was dissected out, and tissue lysates were prepared using RIPA buffer containing protease inhibitor cocktail and 1 mM PMSF. Protein concentrations of supernatant were determined, after lysates were centrifuged for 10 min at 10,000 g. 100 μg of protein from tissue extract was resolved on 10% SDS-PAGE and transferred to PVDF membranes (Millipore, Bedford, MA) with the help of Wet/Tank Blotting System (Bio-Rad Laboratories Inc, Hercules, CA, USA). The membranes were first incubated overnight at 4 °C with earthworm protein antibody (at 1:1000 dilutions) raised in mice. This was followed by the addition of the secondary antibody (1:2000) raised in rabbit and conjugated with alkaline phosphatase. The protein bands were then detected by using 5-bromro 4-chloro 3-indolyl phosphate/nitrobluetetrazolium (BCIP/NBT).

#### Statistical analysis

We performed the two-way ANOVA with three observations per cell to accommodate the temporal variations in the data for CR, C/N ratio, FAC, HAC, DoH, and various fractions of metals (Fe, Zn, Pb, Cr, Cd, and As). In addition, we conducted the tests for the least significant difference (LSD) to identify the real differences between various feed mixtures. The Pearson’s correlation statistics were also performed to assess the relationship between MT content in the earthworm gut and the metal concentration in the feed stocks.

## Additional Information

**How to cite this article**: Goswami, L. *et al*. Exploring metal detoxification and accumulation potential during vermicomposting of Tea factory coal ash: sequential extraction and fluorescence probe analysis. *Sci. Rep.* **6**, 30402; doi: 10.1038/srep30402 (2016).

## Supplementary Material

Supplementary Information

## Figures and Tables

**Table 1:** Changes in C/N ratio and compost respiration and during the period of biocomposting.

Treatments	C:N ratio			Compost resp. (mg g^−1^)		
	0 D	30 D	60 D	0 D	30 D	60 D
TFCA (only)(E1)	4.76 ± 0.10	2.31 ± 0.19	1.07 ± 0.05	0.10 ± 0.08	0.61 ± 0.02	0.684 ± 0.11
TFCA + CD (1:1)(E2)	9.35 ± 0.89	3.21 ± 0.08	6.77 ± 0.15	1.09 ± 0.03	3.96 ± 0.2	4.245 ± 0.28
TFCA + CD (1:2)(E3)	11.24 ± 0.3	4.78 ± 0.56	3.21 ± 0.48	0.90 ± 0.03	3.95 ± 0.53	3.36 ± 0.21
CD(E4)	17.85 ± 1.9	4.14 ± 0.40	7.50 ± 0.26	1.01 ± 0.1	5.26 ± 0.26	4.30 ± 0.26
TFCA(L1)	7.85 ± 0.13	3.13 ± 0.13	3.68 ± 0.19	0.11 ± 0.02	0.51 ± 0.03	0.52 ± 0.05
TFCA + CD (1:1)(L2)	9.64 ± 0.65	3.91 ± 0.14	2.5 ± 0.08	1.24 ± 0.2	3.03 ± 0.3	4.06 ± 0.2
TFCA + CD (1:2)(L3)	13.03 ± 0.7	4.18 ± 0.45	3.81 ± 0.13	1.37 ± 0.09	8.04 ± 0.81	7.55 ± 0.51
CD(L4)	18.57 ± 1.1	2.71 ± 0.08	12.43 ± 0.21	1.15 ± 0.15	6.56 ± 0.71	5.09 ± 0.74
TFCA (only) (C1)	8.26 ± 0.47	3.49 ± 0.07	3.6 ± 0.19	0.11 ± 0.05	0.46 ± 0.055	0.56 ± 0.021
TFCA + CD (1:1)(C2)	7.28 ± 0.12	3.00 ± 0.1	3.1 ± 0.11	1.46 ± 0.2	2.47 ± 0.2	2.84 ± 0.264
TFCA + CD (1:2) (C3)	10.41 ± 0.8	7.07 ± 0.09	6.5 ± 0.31	1.18 ± 0.2	2.07 ± 0.42	3.63 ± 0.32
CD (C4)	16.21 ± 1.7	6.32 ± 0.04	5.8 ± 0.19	1.93 ± 0.1	2.69 ± 0.25	3.85 ± 0.21
P value						
Treatment	0.000			0.000		
Day	0.000			0.000		
Treatment × Day	0.000			0.000		
LSD	1.444			0.017		

**Table 2:** Changes in fulvic acid carbon (FAC), humic acid carbon (HAC), and degree of humification (DoH) during the period of biocomposting.

Treatments	Fulvic Acid Carbon (FAC) (%)			Humic Acid Carbon (HAC) (%)			DoH		
	0 D	30 D	60 D	0 D	30 D	60 D	0 D	30 D	60 D
TFCA (only)(E1)	0.044 ± 0.01	0.023 ± 0.003	0.06 ± 0.002	0.22 ± 0.015	0.075 ± 0.006	0.07 ± 0.002	0.42 ± 0.01	1.08 ± 0.03	1.28 ± 0.072
TFCA + CD (1:1)(E2)	0.011 ± 0.001	0.027 ± 0.003	0.07 ± 0.004	0.26 ± 0.015	0.15 ± 0.02	0.50 ± 0.021	0.47 ± 0.02	2.20 ± 0.02	2.01 ± 0.05
TFCA + CD (1:2)(E3)	0.62 ± 0.038	0.47 ± 0.03	1.08 ± 0.036	0.25 ± 0.015	0.15 ± 0.02	0.51 ± 0.02	0.48 ± 0.02	2.24 ± 0.06	2.06 ± 0.025
CD(E4)	0.55 ± 0.02	0.48 ± 0.055	0.66 ± 0.01	0.18 ± 0.006	0.14 ± 0.021	0.32 ± 0.032	0.50 ± 0.02	2.06 ± 0.02	2.82 ± 0.021
TFCA(L1)	0.024 ± 0.006	0.18 ± 0.025	0.32 ± 0.026	0.08 ± 0.006	0.015 ± 0.001	0.09 ± 0.007	0.33 ± 0.01	1.13 ± 0.02	1.65 ± 0.042
TFCA + CD (1:1)(L2)	0.024 ± 0.006	0.36 ± 0.095	0.64 ± 0.035	0.20 ± 0.01	0.015 ± 0.003	0.35 ± 0.02	0.42 ± 0.01	1.95 ± 0.03	2.39 ± 0.065
TFCA + CD (1:2)(L3)	0.18 ± 0.021	0.61 ± 0.015	0.66 ± 0.026	0.30 ± 0.006	0.12 ± 0.03	0.37 ± 0.030	0.48 ± 0.02	2.23 ± 0.02	2.81 ± 0.017
CD(L4)	0.39 ± 0.02	0.88 ± 0.053	0.60 ± 0.04	0.19 ± 0.02	0.26 ± 0.025	0.47 ± 0.01	0.45 ± 0.01	2.53 ± 0.02	3.59 ± 0.042
TFCA (only)(C1)	0.23 ± 0.031	0.22 ± 0.025	0.21 ± 0.03	0.12 ± 0.006	0.045 ± 0.001	0.026 ± 0.001	0.20 ± 0.07	0.291 ± 0.03	0.44 ± 0.026
TFCA + CD (1:1)(C2)	0.08 ± 0.006	0.35 ± 0.03	0.23 ± 0.015	0.11 ± 0.006	0.23 ± 0.031	0.11 ± 0.007	0.46 ± 0.02	1.30 ± 0.02	2.02 ± 0.047
TFCA + CD (1:2) (C3)	0.27 ± 0.035	0.69 ± 0.055	0.57 ± 0.031	0.21 ± 0.006	0.19 ± 0.017	0.11 ± 0.002	0.47 ± 0.02	1.46 ± 0.04	2.06 ± 0.087
CD(C4)	0.29 ± 0.020	0.96 ± 0.062	0.56 ± 0.035	0.20 ± 0.006	0.23 ± 0.031	0.25 ± 0.002	0.51 ± 0.02	2.32 ± 0.02	2.08 ± 0.025
P value									
Treatment	0.000			0.000				0.000	
Day	0.000			0.000				0.000	
Treatment × Day	0.000			0.000				0.000	
LSD	0.303			0.12				0.16	

**Table 3 t3:** 

*Eisenia fetida* system	*Lampito mauritii* system	Aerobic composting system
E1- Only TFCA	L1- Only TFCA	C1- Only TFCA
E2- TFCA + CD (1:1)	L2- TFCA + CD (1:1)	C2- TFCA + CD (1:1)
E3- TFCA + CD (1:2)	L3- TFCA + CD (1:2)	C3- TFCA + CD (1:2)
E4- Only CD	L4- CD	C4- CD
