# Supplementary material for: Exploring metal detoxification and accumulation potential during vermicomposting of Tea factory coal ash: sequential extraction and fluorescence probe analysis
Source: Sci Rep. 2016 Jul 26;6:30402. doi: 10.1038/srep30402 (PMC4960643; doi:10.1038/srep30402)
Supplement: Supplementary Information [file srep30402-s1.doc]

**Exploring metal detoxification and accumulation potential during vermicomposting of Tea factory coal ash: sequential extraction and fluorescence probe analysis**

Linee Goswami^a^, Sanjay Pratihar^b^, Suman Dasgupta^c^, Pradip Bhattacharyya^d^, Pronab Mudoi^c^, Jayanta Bora^a^ , Satya Sundar Bhattacharya^a^ *, Ki Hyun Kim^e+^

^a^Department of Environmental Science, Tezpur University, Assam 784028, India

^b^Department of Chemical Sciences, Tezpur University, Assam 784028, India

^c^Department of Molecular Biology and Biotechnology, Tezpur University, Assam 784028, India

^d^ Agricultural and Ecological Research Unit, Indian Statistical Institute, Giridih, Jharkhand 815301, India

^e^ Department of Civil & Environmental Engineering, Hanyang University, 222Wangsimni-Ro, Seoul 133-791, Republic of Korea

**Table 1S a: Changes in exchangeable, carbonate, and oxide bound fractions of Zn under various treatments during vermicomposting (mean ± standard deviation).**

| Zn | Exchangeable (mg kg^-1^) | | | Carbonate Bound (mg kg^-1^) | | | Fe Mn Oxide (mg kg^-1^) | | |
| --- | --- | --- | --- | --- | --- | --- | --- | --- | --- |
|  | 0 D | 30 D | 60 D | 0 D | 30 D | 60 D | 0 D | 30 D | 60 D |
| E1 | 0.6±0.01 | 0.045±0.009 | 0.085±0.01 | 0.92±0.03 | 0.36±0.05 | 0.06±0.01 | 1.71±0.11 | 1.89±0.21 | 1.27±0.11 |
| E2 | 0.75±0.12 | 0.45±0.011 | 0.15±0.02 | 0.55±0.07 | 0.15±0.04 | 0.15±0.01 | 0.56±0.06 | 0.31±0.01 | 0.03±0.03 |
| E3 | 0.3±0.06 | 0.45±0.01 | 0.135±0.01 | 0.26±0.09 | 0.075±0.03 | 0.08±0.01 | 0.11±0.02 | 0.03±0.07 | 0.045±0.02 |
| E4 | 0.095±0.08 | 0.045±0.01 | 0.055±0.01 | 0.14±0.06 | 0.09±0.01 | 0.03±0.02 | 1.5±0.31 | 0.3±0.05 | 0.405±0.05 |
| L1 | 0.31±0.09 | 0.15±0.01 | 0.045±0.01 | 0.62±0.06 | 0.12±0.02 | 0.26±0.01 | 0.95±0.07 | 0.78±0.06 | 0.62±0.01 |
| L2 | 0.34±0.06 | 0.05±0.01 | 0.06±0.01 | 0.75±0.08 | 0.135±0.01 | 0.15±0.01 | 0.75±0.06 | 0.45±0.04 | 1.50±0.02 |
| L3 | 0.14±0.04 | 0.02±0.01 | 0.09±0.01 | 0.12±0.02 | 0.075±0.05 | 0.09±0.01 | 0.3±0.02 | 0.015±0.01 | 0.15±0.01 |
| L4 | 0.12±0.03 | 0.09±0.01 | 0.045±0.03 | 3.00±0.03 | 1.35±0.06 | 0.91±0.01 | 0.15±0.012 | 0.09±0.01 | 0.08±0.01 |
| C1 | 0.065±0.02 | 0.045±0.01 | 0.33±0.02 | 0.92±0.07 | 0.05±0.01 | 0.02±0.01 | 1.94±0.14 | 0.67±0.01 | 1.5±0.01 |
| C2 | 0.075±0.03 | 0.03±0.01 | 0.75±0.04 | 0.15±0.04 | 0.09±0.08 | 0.06±0.01 | 0.89±0.08 | 0.57±0.05 | 0.49±0.02 |
| C3 | 0.095±0.05 | 0.045±0.03 | 0.50±0.07 | 0.06±0.02 | 0.05±0.01 | 0.05±0.01 | 0.93±0.09 | 0.46±0.06 | 0.83±0.03 |
| C4 | 0.045±0.003 | 0.06±0.01 | 0.11±0.09 | 0.90±0.06 | 0.45±0.07 | 0.75±0.01 | 1.50±0.31 | 0.11±0.02 | 2.25±0.37 |
| P value |  |  |  |  |  |  |  |  |  |
| Treatment | 0.000 |  |  | 0.000 |  |  | 0.000 |  |  |
| Day | 0.000 |  |  | 0.000 |  |  | 0.000 |  |  |
| Treatment*day | 0.000 |  |  | 0.000 |  |  | 0.000 |  |  |
| LSD | 0.054 |  |  | 0.012 |  |  | 0.034 |  |  |

**Table 1S b: Changes in organic matter and residual bound fractions of Zn under various treatments during vermicomposting (mean ± standard deviation).**

| Zn | Organic matter (mg kg^-1^) | | | Residual (mg kg^-1^) | | |
| --- | --- | --- | --- | --- | --- | --- |
|  | 0 D | 30 D | 60 D | 0 D | 30 D | 60 D |
| E1 | 2.45±0.12 | 2.64±0.19 | 2.58±0.45 | 6.15±0.65 | 7.40±0.78 | 7.95±0.89 |
| E2 | 2.63±0.25 | 2.04±0.12 | 2.60±0.56 | 6.00±0.45 | 10.50±0.83 | 2.40±0.12 |
| E3 | 3.79±0.45 | 3.12±0.31 | 3.33±0.31 | 4.80±0.45 | 1.50±0.21 | 3.15±0.54 |
| E4 | 4.06±0.78 | 2.36±0.26 | 1.69±0.15 | 3.45±0.34 | 5.10±0.65 | 4.80±0.39 |
| L1 | 2.56±0.12 | 3.57±0.36 | 2.67±0.22 | 9.75±0.11 | 12.60±1.02 | 8.55±0.67 |
| L2 | 3.99±0.31 | 3.45±0.41 | 3.90±0.41 | 8.10±0.46 | 9.45±0.77 | 6.90±0.43 |
| L3 | 1.26±0.13 | 3.37±0.48 | 5.01±0.55 | 16.20±0.91 | 12.90±0.78 | 9.30±0.54 |
| L4 | 2.22±0.19 | 2.31±0.15 | 3.51±0.29 | 9.15±0.89 | 14.70±0.31 | 1.50±0.12 |
| C1 | 5.44±0.78 | 1.78±0.16 | 6.54±0.58 | 9.60±0.76 | 11.55±0.94 | 10.31±0.03 |
| C2 | 3.28±0.56 | 2.85±0.28 | 5.19±0.43 | 8.30±0.81 | 12.90±0.41 | 13.35±0.33 |
| C3 | 2.74±0.17 | 1.32±0.09 | 6.72±0.41 | 9.20±0.78 | 12.15±0.42 | 12.15±0.37 |
| C4 | 1.34±0.11 | 2.21±0.27 | 1.85±0.21 | 8.50±0.65 | 9.00±0.58 | 11.25±0.31 |
| P value |  |  |  |  |  |  |
| Treatment | 0.000 |  |  | 0.000 |  |  |
| Day | 0.000 |  |  | 0.000 |  |  |
| Treatment*day | 0.000 |  |  | 0.000 |  |  |
| LSD | 0.034 |  |  | 0.474 |  |  |

**Table 1S c: Changes in exchangeable, carbonate, and oxide bound fractions of Cr under various treatments during vermicomposting (mean ± standard deviation).**

| Cr | Exchangeable (mg kg^-1^) | | | Carbonate Bound (mg kg^-1^) | | | Fe Mn Oxide (mg kg^-1^) | | |
| --- | --- | --- | --- | --- | --- | --- | --- | --- | --- |
|  | 0 D | 30 D | 60 D | 0 D | 30 D | 60 D | 0 D | 30 D | 60 D |
| E1 | 0.30±0.03 | 0.15±0.03 | 0.15±0.04 | 0.78±0.03 | 0.72±0.05 | 0.28±0.02 | 2.04±0.21 | 2.28±0.24 | 1.58±0.13 |
| E2 | 0.45±0.02 | 0.15±0.02 | 0.14±0.03 | 0.14±0.04 | 0.16±0.01 | 0.09±0.02 | 0.95±0.06 | 0.74±0.06 | 0.35±0.06 |
| E3 | 0.15±0.04 | 0.12±0.05 | 0.09±0.03 | 0.03±0.02 | 0.03±0.01 | 0.015±0.01 | 0.38±0.04 | 0.52±0.04 | 0.32±0.03 |
| E4 | 0.30±0.07 | 0.23±0.04 | 0.15±0.03 | 0.03±0.01 | 0.015±0.01 | 0.015±0.02 | 0.12±0.09 | 0.14±0.02 | 0.11±0.09 |
| L1 | 0.36±0.04 | 0.18±0.02 | 0.05±0.01 | 0.54±0.05 | 0.28±0.04 | 0.24±0.01 | 1.40±0.11 | 1.08±0.54 | 0.95±0.06 |
| L2 | 0.31±0.05 | 0.09±0.05 | 0.08±0.01 | 0.11±0.07 | 0.14±0.05 | 0.07±0.01 | 1.31±0.14 | 1.06±0.67 | 0.49±0.03 |
| L3 | 0.28±0.01 | 0.015±0.06 | 0.11±0.02 | 0.30±0.02 | 0.03±0.02 | 0.06±0.01 | 0.41±0.06 | 0.48±0.04 | 0.32±0.02 |
| L4 | 0.19±0.04 | 0.015±0.03 | 0.05±0.01 | 0.09±0.01 | 0.02±0.04 | 0.02±0.01 | 0.25±0.03 | 0.29±0.03 | 0.15±0.06 |
| C1 | 0.05±0.04 | 0.09±0.05 | 0.50±0.01 | 0.86±0.06 | 0.36±0.05 | 0.44±0.06 | 2.28±0.21 | 0.96±0.08 | 1.83±0.11 |
| C2 | 0.45±0.02 | 0.60±0.03 | 0.90±0.05 | 0.15±0.01 | 0.15±0.01 | 0.08±0.03 | 1.41±0.16 | 1.03±0.07 | 0.88±0.08 |
| C3 | 0.11±0.06 | 0.05±0.02 | 0.06±0.01 | 0.08±0.01 | 0.015±0.01 | 0.11±0.07 | 0.73±0.03 | 0.63±0.05 | 0.49±0.05 |
| C4 | 0.02±0.01 | 0.02±0.01 | 0.05±0.01 | 0.02±0.01 | 0.03±0.01 | 0.05±0.03 | 0.19±0.01 | 0.12±0.04 | 0.12±0.02 |
| P value |  |  |  |  |  |  |  |  |  |
| Treatment | 0.000 |  |  | 0.000 |  |  | 0.000 |  |  |
| Day | 0.000 |  |  | 0.000 |  |  | 0.000 |  |  |
| Treatment*day | 0.000 |  |  | 0.000 |  |  | 0.000 |  |  |
| LSD | 1.06 |  |  | 0.013 |  |  | 0.027 |  |  |

**Table 1S d: Changes in organic matter and residual bound fractions of Cr under various treatments during vermicomposting (mean ± standard deviation).**

| Cr | Organic matter (mg kg^-1^) | | | Residual (mg kg^-1^) | | |
| --- | --- | --- | --- | --- | --- | --- |
|  | 0 D | 30 D | 60 D | 0 D | 30 D | 60 D |
| E1 | 2.67±0.11 | 2.70±0.31 | 2.85±0.23 | 6.45±0.55 | 9.75±1.03 | 6.75±0.61 |
| E2 | 2.88±0.45 | 2.19±0.67 | 2.85±0.33 | 7.35±0.67 | 7.95±0.84 | 3.30±0.23 |
| E3 | 4.20±0.56 | 3.40±0.17 | 3.61±0.81 | 6.00±0.78 | 4.50±0.34 | 3.15±0.26 |
| E4 | 2.95±0.41 | 2.61±0.26 | 2.82±0.21 | 4.35±0.38 | 5.55±0.51 | 4.05±0.39 |
| L1 | 2.95±0.31 | 3.76±0.43 | 4.80±0.51 | 10.95±1.14 | 14.10±1.23 | 9.75±0.89 |
| L2 | 4.50±0.39 | 3.55±0.37 | 6.00±0.59 | 8.80±0.78 | 9.60±0.82 | 6.60±0.56 |
| L3 | 1.35±0.13 | 3.66±0.27 | 3.99±0.42 | 10.10±1.01 | 15.75±1.23 | 10.30±1.02 |
| L4 | 2.46±0.21 | 2.59±0.36 | 4.08±0.73 | 6.65±0.89 | 17.85±2.32 | 3.30±0.44 |
| C1 | 5.92±0.67 | 2.07±0.54 | 7.47±0.88 | 11.10±1.02 | 13.50±2.04 | 14.60±0.99 |
| C2 | 3.24±0.82 | 3.19±0.34 | 5.82±0.49 | 11.80±0.82 | 14.40±0.68 | 15.45±1.05 |
| C3 | 3.05±0.24 | 1.38±0.29 | 7.57±1.04 | 11.70±0.22 | 14.40±0.23 | 14.25±0.54 |
| C4 | 1.93±0.17 | 2.50±0.21 | 2.43±0.22 | 5.30±0.51 | 10.50±0.42 | 10.75±0.59 |
| P value |  |  |  |  |  |  |
| Treatment | 0.000 |  |  | 0.000 |  |  |
| Day | 0.000 |  |  | 0.000 |  |  |
| Treatment*day | 0.000 |  |  | 0.000 |  |  |
| LSD | 0.027 |  |  | 0.539 |  |  |

**Table 1S e: Changes in different fractions of Cd under various treatments during vermicomposting (mean ± standard deviation).**

| Cd | Exchangeable (mg kg^-1^) | | | Carbonate Bound (mg kg^-1^) | | | Fe Mn Oxide (mg kg^-1^) | | | Organic bound | | | Residual | | | | | | |  | |
| --- | --- | --- | --- | --- | --- | --- | --- | --- | --- | --- | --- | --- | --- | --- | --- | --- | --- | --- | --- | --- | --- |
|  | 0 D | 30 D | 60 D | 0 D | 30 D | 60 D | 0 D | 30 D | 60 D | 0 D | 30 D | 60 D | 0 D | 30 D | | | 60 D | | |  | |
| E1 | 3.50±0.22 | 2.25±0.45 | 2.10±0.31 | 6.09±0.30 | 4.34±0.17 | 3.50±0.15 | 2.55±0.30 | 2.70±0.15 | 2.30±0.23 | BDL* | BDL | BDL | BDL | | | BDL | | | BDL | |  |
| E2 | 1.90±0.22 | 2.35±0.90 | 1.60±0.37 | 5.78±0.41 | 4.83±0.12 | 2.15±0.45 | 3.50±0.37 | 3.70±0.53 | 3.35±0.23 | BDL | BDL | BDL | BDL | | BDL | | | BDL | | |  |
| E3 | 2.28±0.18 | 2.75±0.90 | 1.25±0.22 | 6.46±0.34 | 4.87±0.12 | 2.96±0.18 | 4.30±0.45 | 4.35±0.30 | 4.35±0.15 | BDL | BDL | BDL | BDL | | BDL | | | BDL | | |  |
| E4 | 2.00±0.68 | 3.15±0.22 | 1.75±0.70 | 6.86±0.43 | 6.485±0.12 | 6.13±0.27 | 5.05±0.60 | 8.35±0.22 | 4.50±0.52 | BDL | BDL | BDL | BDL | | | BDL | | | BDL | |  |
| L1 | 3.90±0.48 | 1.60±0.22 | 2.35±0.39 | 7.03±0.33 | 4.57±0.11 | 5.29±0.22 | 4.85±0.37 | 4.9±0.15 | 4.70±0.30 | BDL | BDL | BDL | BDL | | BDL | | | BDL | | | |
| L2 | 2.10±0.39 | 2.40±0.39 | 2.05±0.83 | 4.70±0.31 | 4.61±0.05 | 3.45±0.26 | 3.75±0.60 | 3.45±0.30 | 3.70±0.62 | BDL | BDL | BDL | BDL | | | BDL | | | BDL |  | |
| L3 | 2.05±0.56 | 2.60±0.56 | 1.90±0.22 | 7.52±0.17 | 4.78±0.03 | 2.66±0.18 | 4.35±0.54 | 3.60±0.45 | 5.20±0.48 | BDL | BDL | BDL | BDL | | | BDL | | | BDL |  | |
| L4 | 2.35±0.60 | 2.60±0.83 | 1.35±0.60 | 5.72±0.17 | 5.22±0.24 | 1.90±0.51 | 2.05±0.52 | 2.70±0.37 | 2.10±0.37 | BDL | BDL | BDL | BDL | | BDL | | | BDL | | | |
| C1 | 5.45±0.36 | 4.55±0.45 | 6.35±0.15 | 7.62±0.58 | 4.67±0.04 | 5.71±0.22 | 4.95±0.45 | 3.10±0.54 | 6.50±0.15 | BDL | BDL | BDL | BDL | | | BDL | | | BDL |  | |
| C2 | 2.10±0.39 | 3.85±0.45 | 5.15±0.30 | 6.19±0.06 | 5.10±0.14 | 1.92±0.06 | 3.75±0.54 | 4.30±0.39 | 7.40±0.52 | BDL | BDL | BDL | BDL | | | BDL | | | BDL |  | |
| C3 | 3.12±0.36 | 2.30±0.62 | 3.40±0.22 | 8.43±0.24 | 6.61±0.35 | 3.14±0.22 | 4.35±0.39 | 5.85±0.30 | 8.75±0.30 | BDL | BDL | BDL | BDL | | | BDL | | | BDL |  | |
| C4 | 2.35±0.3 | 3.10±0.90 | 4.35±0.97 | 5.63±0.15 | 4.76±0.25 | 2.65±0.62 | 2.40±0.99 | 3.80±0.60 | 4.45±0.31 | BDL | BDL | BDL | BDL | | | BDL | | | BDL |  | |
| P value |  |  |  |  |  |  |  |  |  |  |  |  |  |  | | |  | | | | |
| Treatment | 0.000 |  |  | 0.000 |  |  | 0.000 |  |  |  |  |  |  |  | | |  | | | | |
| Day | 0.000 |  |  | 0.000 |  |  | 0.000 |  |  |  |  |  |  |  | | |  | | | | |
| Treatment*day | 0.000 |  |  | 0.000 |  |  | 0.000 |  |  |  |  |  |  |  | | |  | | | | |
| LSD | 0.25 |  |  | 0.134 |  |  | 0.209 |  |  |  |  |  |  |  | | |  | | | | |

***Detection limit for Cd in the instrument (ICP-OES) was 0.02 mg kg^-1^**

**Table 1S f: Changes in exchangeable fraction of As under various treatments during vermicomposting (mean ± standard deviation).**

| As | Exchangeable (mg kg^-1^) | |  |
| --- | --- | --- | --- |
|  | 0 D | 30 D | 60 D |
| E1 | 0.15±0.02 | 0.39±0.02 | 0.49±0.01 |
| E2 | 0.45±0.06 | 0.31±0.02 | 0.06±0.01 |
| E3 | 0.81±0.07 | 0.79±0.02 | 0.57±0.04 |
| E4 | 0.84±0.01 | 0.61±0.02 | 0.77±0.03 |
| L1 | 0.43±0.05 | 0.39±0.01 | 0.16±0.01 |
| L2 | 1.84±0.06 | 0.58±0.03 | 0.33±0.04 |
| L3 | 1.88±0.18 | 0.27±0.01 | 0.66±0.11 |
| L4 | 0.31±0.01 | 0.27±0.03 | 0.18±0.03 |
| C1 | 0.028±0.01 | 0.044±0.01 | 0.72±0.09 |
| C2 | 0.49±0.06 | 0.87±0.05 | 1.44±0.05 |
| C3 | 0.13±0.02 | 0.61±0.03 | 1.03±0.01 |
| C4 | 0.49±0.06 | 0.61±0.07 | 0.91±0.02 |
| P value |  |  |  |
| Treatment | 0.000 |  |  |
| Day | 0.000 |  |  |
| Treatment*day | 0.000 |  |  |
| LSD | 0.03 |  |  |

**Table 1S g: Changes in exchangeable, carbonate, and oxide bound fractions of Fe under various treatments during vermicomposting (mean ± standard deviation).**

| Fe | Exchangeable (mg kg^-1^) | | | Carbonate Bound (mg kg^-1^) | | | Fe Mn Oxide (mg kg^-1^) | |  |
| --- | --- | --- | --- | --- | --- | --- | --- | --- | --- |
|  | 0 D | 30 D | 60 D | 0 D | 30 D | 60 D | 0 D | 30 D | 60 D |
| E1 | 0.42±0.03 | 0.41±0.06 | 0.32±0.08 | 12.65±0.23 | 23.82±2.11 | 9.32±0.89 | 917.02±43.21 | 1168.12±79.07 | 859.20±94.12 |
| E2 | 0.82±0.06 | 0.49±0.08 | 0.66±0.15 | 3.90±0.07 | 13.41±1.45 | 3.48±0.23 | 591.37±31.32 | 866.62±42.12 | 293.85±30.11 |
| E3 | 1.01±0.08 | 1.47±0.21 | 0.41±0.07 | 1.08±0.054 | 1.57±0.21 | 1.75±0.09 | 273.09±19.89 | 327.00±30.01 | 264.23±19.89 |
| E4 | 1.87±0.21 | 0.95±0.11 | 0.54±0.09 | 3.24±0.32 | 0.83±0.05 | 2.04±0.31 | 215.32±15.67 | 164.76±11.56 | 146.13±12.04 |
| L1 | 1.94±0.19 | 4.50±0.08 | 6.00±0.61 | 11.82±1.01 | 11.64±1.01 | 15.51±1.67 | 868.50±8.98 | 1069.80±78.43 | 571.80±34.23 |
| L2 | 0.37±0.04 | 2.68±0.09 | 0.41±0.05 | 2.45±0.24 | 6.24±0.67 | 4.39±0.34 | 815.40±54.21 | 633.75±56.21 | 397.95±31.22 |
| L3 | 1.09±0.06 | 0.26±0.01 | 2.92±0.98 | 1.44±0.07 | 1.77±0.04 | 5.89±0.55 | 394.35±23.21 | 422.85±39.81 | 324.75±24.88 |
| L4 | 1.99±0.31 | 0.83±0.07 | 4.75±0.56 | 3.44±0.45 | 1.96±0.06 | 0.87±0.06 | 434.25±31.23 | 245.7±21.12 | 273.15±27.06 |
| C1 | 4.23±0.42 | 0.72±0.06 | 1.43±0.21 | 12.75±0.67 | 13.12±1.06 | 11.15±1.48 | 1235.40±78.82 | 1170.15±89.91 | 1163.55±91.23 |
| C2 | 0.48±0.06 | 1.14±0.087 | 0.35±0.09 | 6.55±0.51 | 8.04±0.67 | 3.03±0.65 | 964.65±65.21 | 642.75±45.32 | 1088.85±89.32 |
| C3 | 1.11±0.08 | 0.02±0.006 | 2.92±0.54 | 2.15±0.17 | 2.10±0.045 | 7.31±0.89 | 488.62±39.89 | 582.82±31.89 | 479.85±39.89 |
| C4 | 1.62±0.17 | 3.48±0.87 | 1.29±0.16 | 4.53±0.34 | 6.76±0.67 | 2.95±0.23 | 425.70±27.75 | 270.00±21.12 | 202.05±19.65 |
| P value |  |  |  |  |  |  |  |  |  |
| Treatment | 0.000 |  |  | 0.000 |  |  | 0.000 |  |  |
| Day | 0.000 |  |  | 0.000 |  |  | 0.000 |  |  |
| Treatment*day | 0.000 |  |  | 0.000 |  |  | 0.000 |  |  |
| LSD | 0.0938 |  |  | 0.2618 |  |  | 12.74 |  |  |

**Table 1S h Changes in organic matter and residual bound fractions of Fe under various treatments during vermicomposting (mean ± standard deviation).**

| Fe | Organic Matter (mg kg^-1^) | | | Residual (mg kg^-1^) | | |
| --- | --- | --- | --- | --- | --- | --- |
|  | 0 D | 30 D | 60 D | 0 D | 30 D | 60 D |
| E1 | 346.95±27.78 | 387.00±19.12 | 997.43±78.18 | 2641.50±123.45 | 2865.00±231.45 | 2785.50±115.45 |
| E2 | 825.00±56.34 | 1659.00±65.45 | 727.73±54.21 | 3402.00±267.44 | 2025.00±167.55 | 3186.00±213.45 |
| E3 | 1052.70±67.43 | 295.05±15.55 | 581.10±35.31 | 3016.50±178.43 | 6780.00±345.56 | 9831.00±455.21 |
| E4 | 1168.50±45.23 | 594.75±23.31 | 785.25±45.55 | 2509.50±198.34 | 2662.35±222.54 | 2818.50±145.23 |
| L1 | 365.40±21.16 | 1262.71±11.67 | 1641.00±67.78 | 2697.00±202.34 | 4842.00±305.45 | 5163.00±298.67 |
| L2 | 435.90±17.81 | 800.41±35.56 | 605.25±34.12 | 3702.00±188.45 | 4234.50±232.78 | 3862.50±189.34 |
| L3 | 171.15±12.14 | 784.50±32.12 | 1451.40±101.05 | 4222.50±267.55 | 6691.50±311.25 | 5296.50±265.21 |
| L4 | 590.85±21.33 | 297.30±25.56 | 1665.60±123.21 | 3628.50±168.89 | 5359.50±239.45 | 23.10±1.89 |
| C1 | 672.75±49.78 | 430.35±34.78 | 2125.50±133.21 | 3421.50±166.67 | 5424.00±244.56 | 308.40±21.45 |
| C2 | 526.35±36.76 | 514.05±49.89 | 5142.00±201.23 | 5286.00±232.31 | 4644.00±378.45 | 17040.00±566.78 |
| C3 | 837.31±81.11 | 1116.60±89.67 | 3291.00±187.23 | 2877.00±121.11 | 11982.00±666.31 | 9583.50±345.21 |
| C4 | 339.15±22.12 | 470.25±37.78 | 1307.70±77.54 | 7345.50±265.11 | 3816.00±134.21 | 3958.50±178.87 |
| P value |  |  |  |  |  |  |
| Treatment | 0.000 |  |  | 0.000 |  |  |
| Day | 0.000 |  |  | 0.000 |  |  |
| Treatment*day | 0.000 |  |  | 0.000 |  |  |
| LSD | 12.74 |  |  | 11.98 |  |  |

**Table 1S i: Changes in exchangeable, carbonate, and oxide bound fractions of Pb under various treatments during vermicomposting (mean ± standard deviation).**

| Pb | Exchangeable (mg kg^-1^) | | | Carbonate (mg kg^-1^) | | | Fe Mn Oxide (mg kg^-1^) | | |
| --- | --- | --- | --- | --- | --- | --- | --- | --- | --- |
|  | 0 D | 30 D | 60 D | 0 D | 30 D | 60 D | 0 D | 30 D | 60 D |
| E1 | 0.03±0.01 | 0.015±0.01 | 0.025±0.01 | 0.27±0.03 | 0.12±0.03 | 0.15±0.01 | 0.09±0.03 | 0.12±0.01 | 0.18±0.01 |
| E2 | 0.4±0.01 | 0.3±0.01 | 0.1±0.01 | 0.23±0.01 | 0.12±0.02 | 0.18±0.01 | 0.15±0.02 | 0.15±0.01 | 0.18±0.01 |
| E3 | 0.3±0.01 | 0.015±0.02 | 0.03±0.01 | 0.27±0.01 | 0.23±0.01 | 0.23±0.01 | 0.24±0.02 | 0.20±0.01 | 0.22±0.03 |
| E4 | 0.45±0.01 | 0.015±0.02 | 0.045±0.01 | 0.34±0.02 | 0.17±0.01 | 0.27±0.02 | 0.74±0.03 | 0.54±0.01 | 0.53±0.01 |
| L1 | 0.33±0.01 | 0.015±0.02 | 0.035±0.01 | 0.21±0.01 | 0.19±0.02 | 0.14±0.01 | 0.12±0.02 | 0.09±0.01 | 0.09±0.02 |
| L2 | 0.31±0.01 | 0.15±0.01 | 0.14±0.01 | 0.23±0.01 | 0.27±0.01 | 0.25±0.02 | 0.06±0.01 | 0.16±0.01 | 0.18±0.01 |
| L3 | 0.35±0.005 | 0.18±0.03 | 0.15±0.01 | 0.24±0.01 | 0.28±0.01 | 0.16±0.01 | 0.25±0.02 | 0.28±0.01 | 0.28±0.01 |
| L4 | 0.58±0.02 | 0.39±0.05 | 0.28±0.01 | 0.39±0.01 | 0.32±0.06 | 0.25±0.01 | 1.12±0.06 | 0.59±0.01 | 0.69±0.01 |
| C1 | 0.02±0.01 | 0.09±0.01 | 0.75±0.01 | 0.37±0.01 | 0.31±0.01 | 0.24±0.01 | 0.12±0.03 | 0.19±0.01 | 0.11±0.01 |
| C2 | 0.42±0.02 | 0.24±0.01 | 0.21±0.01 | 0.30±0.01 | 0.19±0.01 | 0.19±0.02 | 0.63±0.05 | 0.46±0.01 | 0.15±0.01 |
| C3 | 0.16±0.02 | 0.14±0.01 | 0.18±0.01 | 0.32±0.01 | 0.25±0.01 | 0.29±0.03 | 0.097±0.01 | 0.23±0.02 | 0.23±0.01 |
| C4 | 0.35±0.02 | 0.45±0.01 | 0.76±0.01 | 0.36±0.01 | 0.24±0.01 | 0.33±0.02 | 1.09±0.02 | 0.52±0.01 | 0.42±0.01 |
| P value |  |  |  |  |  |  |  |  |  |
| Treatment | 0.000 |  |  | 0.000 |  |  | 0.000 |  |  |
| Day | 0.000 |  |  | 0.000 |  |  | 0.000 |  |  |
| Treatment*day | 0.000 |  |  | 0.000 |  |  | 0.000 |  |  |
| LSD | 0.558 |  |  | 0.009 |  |  | 0.012 |  |  |

**Table 1S j: Changes in organic matter and residual bound fractions of Pb under various treatments during vermicomposting (mean ± standard deviation).**

| Pb | Organic matter (mg kg^-1^) | | | Residual (mg kg^-1^) | | |
| --- | --- | --- | --- | --- | --- | --- |
|  | 0 D | 30 D | 60 D | 0 D | 30 D | 60 D |
| E1 | 0.105±0.01 | 0.30±0.01 | 0.65±0.01 | 2.25±0.40 | 1.5±0.03 | 1.05±0.04 |
| E2 | 1.54±0.05 | 1.20±0.08 | 1.50±0.03 | 1.05±0.20 | 3.3±0.03 | 0.45±0.01 |
| E3 | 0.06±0.01 | 0.015±0.03 | 0.015±0.01 | 0.9±0.07 | 0.9±0.01 | 0.15±0.01 |
| E4 | 0.24±0.01 | 0.29±0.01 | 0.12±0.03 | 1.05±0.03 | 0.75±0.01 | 0.60±0.01 |
| L1 | 0.78±0.01 | 0.72±0.01 | 0.51±0.01 | 0.60±0.01 | 0.45±0.01 | 0.30±0.01 |
| L2 | 1.09±0.08 | 1.27±0.31 | 0.30±0.01 | 0.15±0.01 | 1.50±0.12 | 0.15±0.01 |
| L3 | 1.05±0.08 | 0.72±0.06 | 0.63±0.01 | 2.55±0.05 | 0.90±0.01 | 1.05±0.04 |
| L4 | 2.25±0.04 | 1.86±0.05 | 2.54±0.21 | 10.20±1.05 | 1.65±0.01 | 2.85±0.07 |
| C1 | 0.23±0.01 | 0.015±0.03 | 2.72±0.26 | 0.75±0.01 | 0.30±0.01 | 0.23±0.01 |
| C2 | 2.43±0.16 | 2.37±0.03 | 2.31±0.31 | 0.30±0.01 | 0.15±0.02 | 1.65±0.25 |
| C3 | 0.96±0.05 | 0.48±0.01 | 3.23±0.27 | 1.05±0.03 | 1.95±0.03 | 0.45±0.01 |
| C4 | 0.69±0.01 | 0.36±0.01 | 0.32±0.01 | 2.55±0.06 | 1.50±0.01 | 1.35±0.11 |
| P value |  |  |  |  |  |  |
| Treatment | 0.000 |  |  | 0.000 |  |  |
| Day | 0.000 |  |  | NS |  |  |
| Treatment*day | 0.000 |  |  | NS |  |  |
| LSD | 0.01209 |  |  |  |  |  |

**Table 2S: Temporal variation in total concentration of Fe and Zn during the biocomposting process**

| Treatments | Fe(mg kg^-1^) | | | Zn(mg kg^-1^) | | |
| --- | --- | --- | --- | --- | --- | --- |
|  | 0 D | 30 D | 60 D | 0 D | 30 D | 60 D |
| E1 | 3918.54±300.45 | 4444.35±387.5 | 4651.75±504.23 | 10.99±1.11 | 12.34±1.45 | 11.84±0.98 |
| E2 | 4823.09±387.98 | 4564.53±511.55 | 4211.71±389.11 | 10.08±0.92 | 13.45±1.54 | 5.25±0.78 |
| E3 | 4344.37±455.78 | 7405.09±721.44 | 10678.48±1012.5 | 9.12±0.89 | 5.17±0.62 | 6.72±0.83 |
| E4 | 3898.44±278.52 | 3423.63±311.23 | 3752.46±342.45 | 9.19±0.78 | 7.89±0.56 | 7.00±0.68 |
| L1 | 3944.65±407.56 | 7190.64±801.32 | 7397.31±643.27 | 13.68±1.34 | 17.14±1.83 | 11.94±1.23 |
| L2 | 4956.12±501.45 | 5677.57±589.67 | 4870.50±343.67 | 13.74±1.45 | 13.54±1.56 | 12.43±1.42 |
| L3 | 4790.53±453.78 | 7900.87±811.23 | 7081.47±689.67 | 17.89±1.98 | 16.39±1.48 | 14.59±1.23 |
| L4 | 4659.03±399.67 | 5905.29±601.17 | 1967.47±101.42 | 14.61±1.33 | 18.55±1.91 | 6.03±0.54 |
| C1 | 5346.62±511.51 | 7038.35±719.89 | 3610.02±289.67 | 17.91±1.88 | 14.1±1.32 | 8.685±0.98 |
| C2 | 6784.03±611.23 | 5809.98±512.23 | 23274.22±2001.8 | 12.69±1.31 | 16.44±1.45 | 19.54±2.01 |
| C3 | 4206.18±406.76 | 13683.54±1288.95 | 13364.58±1178.6 | 13.07±1.23 | 14.03±1.55 | 20.05±1.99 |
| C4 | 8116.50±767.65 | 4566.49±421.56 | 5472.49±489.99 | 12.28±1.22 | 11.82±1.07 | 11.2±1.05 |
| P value |  |  |  |  |  |  |
| Treatment | 0.003 |  |  | 0.004 |  |  |
| Day | 0.004 |  |  | 0.003 |  |  |
| Treatment×Day | NS |  |  | 0.031 |  |  |
| LSD | 843.22 |  |  | 2.42 |  |  |

**Table 3S: Temporal variation in metal concentration of Cr, Pb, and Cd during the biocomposting process**

| Treatments | Cr (mg kg^-1^) | | | Pb(mg kg^-1^) | | | Cd(mg kg^-1^) | | |
| --- | --- | --- | --- | --- | --- | --- | --- | --- | --- |
|  | 0 D | 30 D | 60 D | 0 D | 30 D | 60 D | 0 D | 30 D | 60 D |
| E1 | 12.25±1.36 | 15.60±1.47 | 11.62±1.02 | 2.75±0.31 | 2.06±0.18 | 2.05±0.19 | 11.63±1.25 | 9.28±0.88 | 5.55±0.07 |
| E2 | 11.76±1.81 | 11.19±1.04 | 6.73±0.58 | 3.24±0.28 | 5.08±0.48 | 2.41±0.19 | 11.18±1.04 | 10.88±1.15 | 7.10±0.68 |
| E3 | 10.76±1.02 | 8.57±0.83 | 7.19±0.98 | 1.77±0.21 | 1.35±0.14 | 0.63±0.07 | 13.04±1.21 | 11.97±1.41 | 8.56±0.87 |
| E4 | 7.75±0.84 | 8.54±0.85 | 7.14±0.84 | 2.82±0.31 | 1.76±0.21 | 1.53±0.17 | 27.91±2.88 | 19.98±2.03 | 18.13±1.76 |
| L1 | 16.21±1.52 | 19.42±2.01 | 15.88±1.67 | 1.74±0.19 | 1.47±0.16 | 1.05±0.12 | 10.12±0.99 | 8.88±0.89 | 5.35±0.56 |
| L2 | 14.86±1.81 | 14.45±1.32 | 13.25±1.13 | 1.84±0.19 | 3.36±0.28 | 1.02±0.09 | 10.55±1.21 | 10.46±1.06 | 9.19±0.93 |
| L3 | 11.92±1.21 | 19.94±2.07 | 16.68±1.87 | 4.44±0.38 | 2.37±0.21 | 2.28±0.25 | 13.92±1.56 | 10.98±1.11 | 9.76±0.95 |
| L4 | 9.39±0.89 | 20.76±1.67 | 7.56±0.81 | 14.55±1.78 | 4.81±0.51 | 6.63±0.71 | 29.98±3.11 | 24.92±2.61 | 20.34±2.76 |
| C1 | 20.21±1.99 | 16.98±1.71 | 24.84±2.55 | 1.49±0.17 | 0.92±0.087 | 4.04±0.38 | 10.37±1.06 | 10.33±1.01 | 11.45±1.01 |
| C2 | 17.05±1.82 | 19.68±2.12 | 22.83±2.31 | 4.08±0.42 | 3.42±0.31 | 4.52±0.46 | 12.05±1.19 | 14.25±1.34 | 14.47±1.58 |
| C3 | 15.66±1.78 | 16.47±1.54 | 22.48±2.31 | 2.58±0.27 | 3.05±0.31 | 4.38±0.42 | 15.91±1.78 | 14.76±1.63 | 15.29±1.61 |
| C4 | 7.46±0.72 | 13.17±1.23 | 13.39±1.42 | 5.04±0.48 | 3.08±0.28 | 3.18±0.30 | 31.01±2.66 | 28.66±3.33 | 32.51±3.45 |
| P value |  |  |  |  |  |  |  |  |  |
| Treatment | 0.021 |  |  |  | 0.003 |  |  | 0.000 |  |
| Day | 0.023 |  |  |  | 0.002 |  |  | 0.001 |  |
| Treatment×Day | NS |  |  |  | 0.042 |  |  | 0.003 |  |
| LSD | 2.223 |  |  |  | 0.722 |  |  | 2.932 |  |

**Table 4S: Pearson's correlation matrix of metallothionein (MT) content in earthworm and metal concentration after 60 days of incubation**

|  | MT | Zn | Cr | Pb | Cd |
| --- | --- | --- | --- | --- | --- |
| MT | 1 |  |  |  |  |
| Zn | 0.41738 | 1 |  |  |  |
| Cr | 0.61335 | 0.95167 | 1 |  |  |
| Pb | -0.1823 | -0.3439 | -0.2795 | 1 |  |
| Cd | -0.3917 | -0.4591 | -0.4675 | 0.65062 | 1 |
